# Implementation of homology based and non-homology based computational methods for the identification and annotation of orphan enzymes: using *Mycobacterium tuberculosis* H37Rv as a case study

**DOI:** 10.1186/s12859-020-03794-x

**Published:** 2020-10-19

**Authors:** Swati Sinha, Andrew M. Lynn, Dhwani K. Desai

**Affiliations:** 1 Bioinformatics Institute, Agency for Science, Technology, and Research (A*Star), 30 Biopolis Street, #07-01 Matrix, Singapore, 138671 Republic of Singapore; 2 Department of Biology and Department of Pharmacology, Dalhousie University, Halifax, NS B3H4R2 Canada; 3 School of Computational and Integrative Sciences, Jawaharlal Nehru University, New Delhi, India

**Keywords:** Homology based method, Non-homology based methods, Local hole, Global hole, Chokepoints, Missing enzyme, Genome context-based annotation, ModEnzA

## Abstract

**Background:**

Homology based methods are one of the most important and widely used approaches for functional annotation of high-throughput microbial genome data. A major limitation of these methods is the absence of well-characterized sequences for certain functions. The non-homology methods based on the context and the interactions of a protein are very useful for identifying missing metabolic activities and functional annotation in the absence of significant sequence similarity. In the current work, we employ both homology and context-based methods, incrementally, to identify local holes and chokepoints, whose presence in the *Mycobacterium tuberculosis* genome is indicated based on its interaction with known proteins in a metabolic network context, but have not been annotated. We have developed two computational procedures using network theory to identify orphan enzymes (‘Hole finding protocol’) coupled with the identification of candidate proteins for the predicted orphan enzyme (‘Hole filling protocol’). We propose an integrated interaction score based on scores from the STRING database to identify candidate protein sequences for the orphan enzymes from *M. tuberculosis*, as a case study, which are most likely to perform the missing function.

**Results:**

The application of an automated homology-based enzyme identification protocol, ModEnzA, on *M. tuberculosis* genome yielded 56 novel enzyme predictions. We further predicted 74 putative local holes, 6 choke points, and 3 high confidence local holes in the genome using ‘Hole finding protocol’. The ‘Hole-filling protocol’ was validated on the *E. coli* genome using artificial in-silico enzyme knockouts where our method showed 25% increased accuracy, compared to other methods, in assigning the correct sequence for the knocked-out enzyme amongst the top 10 ranks. The method was further validated on 8 additional genomes.

**Conclusions:**

We have developed methods that can be generalized to augment homology-based annotation to identify missing enzyme coding genes and to predict a candidate protein for them. For pathogens such as *M. tuberculosis*, this work holds significance in terms of increasing the protein repertoire and thereby, the potential for identifying novel drug targets.

## Background

The function of a protein can be inferred by analyzing its sequence similarity with other proteins of well-characterized functions. In case of significant sequence similarity, the annotation of a protein with a known function is transferred to the protein with an unknown function. These homology-based methods rely on a comparison between two sequences or between a sequence and a profile hidden Markov model (HMM) or between two profile HMMs [1–3]. The most commonly used state of the art approach for finding similarities between two proteins is BLAST [1]. However, there are limitations of this method such as the presence of low complexity regions which can give artificially high scores, significant variation within a protein family, a small number of substrate-specificity determining residues, and discontinuous conserved patterns. To improve these limitations the development of better sensitive methods using sequence-profile comparisons was implemented.

A profile built from multiple sequences is considered to be more sensitive than a single sequence because it incorporates information from multiple sequences. PSI-BLAST [4] is one such method that performs multiple iterations to search for distant homologs in a sequence database and relies on producing a position-specific scoring matrix (PSSM) from them. In such an iterative process, any addition of non-homologous sequences further increases the inclusion of more such non-relevant sequences which in turn reduces the overall specificity of the method. Profile hidden Markov models (HMMs), which contain position-specific probabilities for insertions and deletions along the alignment, perform better than PSSMs for the identification of distant homologs. HMMER [5] is one the most widely used method for searching sequence databases for homologs of protein sequences which utilizes probabilistic models called profile HMMs and is the underlying model building program for the PFAM database [6].

The conservation patterns in a multiple sequence alignment of protein families arise from fold-specific and function-specific signals shared across the entire family and unique to the subfamily level respectively. Since a profile HMM is a probabilistic representation of the alignment, therefore, an HMM built from such an alignment is biased to generate a large number of false-positive (FP) sequences. Methods that use pre-classified data consisting of positive and negative training sets to modify emission and transition probabilities using the Viterbi algorithm were used to increase the specificity of the HMMs [7, 8]. Also, methods relying on positional entropy [9] and Support vector machines (SVMs) [10, 11] were developed to accurately classify sequences by discriminating fold and function-specific signals. On a similar line, HMM-ModE [12, 13], was developed in-house that improves the specificity of prediction by modifying the emission probabilities of the profile HMM model. The method was later implemented on a dataset of known enzymes to develop a novel protocol called ModEnzA [14]. This method can accurately identify metabolic enzymes from completely sequenced genomes and has been shown to perform better than other similar methods like PRIAM [15], MetaShark [16], and EFICAz [17].

However, in case of absence of significant sequence similarity where none of these methods can predict a homolog, genome context-based or non-homology based methods could be used [18]. In general, these methods utilize information such as co-occurrence of proteins involved in the same pathway in a single genome, proximity of such co-occurring genes on the chromosome, or sharing of regulatory sites [18–20]. Protein–protein interactions and co-expression of genes have also been used for assigning function to protein sequences [21, 22]. In terms of enzyme annotation, despite the current advancements in sequencing technology, there are still a significant number of enzymes without any assigned sequences. These enzymes are referred to as orphan enzymes (missing enzymes or holes) [23, 24]. In a metabolic network, orphan enzymes that are lacking an associated protein sequence across any known genome are also referred to as ‘global holes’. On the other hand, a ‘local hole’ has orthologs known but no sequence is known in the species under study. Besides, a ‘chokepoint’ enzyme in a metabolic network is one that is either producing a unique substrate or consuming a unique reactant [25]. In other words, if the metabolic network is visualized as a graph then the choke points are the nodes having one in-degree (number of arcs incident on the vertex) and one out-degree (number of arcs going away from the vertex) (see Fig. 4 [25]). Chokepoint enzymes that are unique to pathogenic genomes can be good candidates for potential drug targets. It is not possible solely from the homology-based approaches to identify the global holes as there is no sequence level information while the local holes and chokepoints may be missed if there is not enough sequence-based homology. Therefore, to overcome this limitation, we propose a metabolic network-based approach to find such missing enzymes. The main focus of this study is to highlight the importance of incremental use of non-homology based methods for enzyme annotation in conjunction with already existing homology-based approaches. First, we utilized ModEnzA [14] to predict all possible enzymes using the sequence level similarities, and secondly, we predicted additional enzymes that are missed by sequence homology-based methods using the protocols developed in this work.

**Fig. 1:**
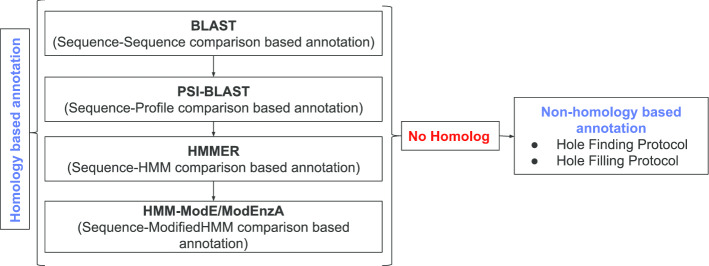
Stepwise systematic approach for the implementation of homology-based and non-homology based computational methods

We proposed two different protocols utilizing information from genome context-based methods for the identification of orphan enzymes referred to as the ‘Hole finding protocol’ as well as identification of candidate proteins for the predicted orphan enzyme referred to as ‘Hole filling protocol’. We used the homology-based method for enzyme annotation i.e. ModEnzA [14] and context-based based ‘Hole finding and filling protocols’, to map the proteome of *Mycobacterium tuberculosis* (referred henceforth as *M. tb*) strain ATCC 25,618 /H37Rv (NCBI Taxonomic ID:83332), in terms of its enzymatic activities. Such a stepwise systematic approach for the implementation of the discussed methods, as shown in Fig. 1, helps to complete the partial genome annotation projects and contribute to identifying novel functions even in the absence of obvious sequence similarities.

## Results

### Identification of enzymes in *M. tb*

**Fig. 2:**
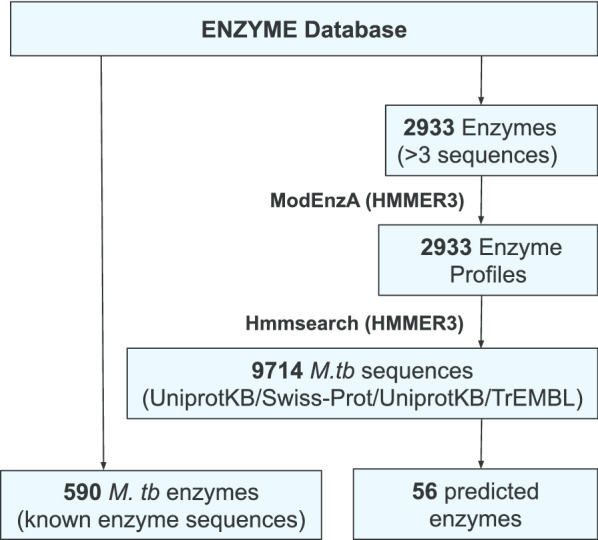
Schematic representation of the workflow to map novel enzymes in *M. tb* using ModEnzA profiles. The ModEnzA enzymes profiles were built with the 31 January 2018 release of the ENZYME database. Both Uniprot-KB/Swiss-Prot and UniProtKB/TrEMBL were used as the sequence search space to scan for novel * M. tb* enzymes

**Fig. 3:**
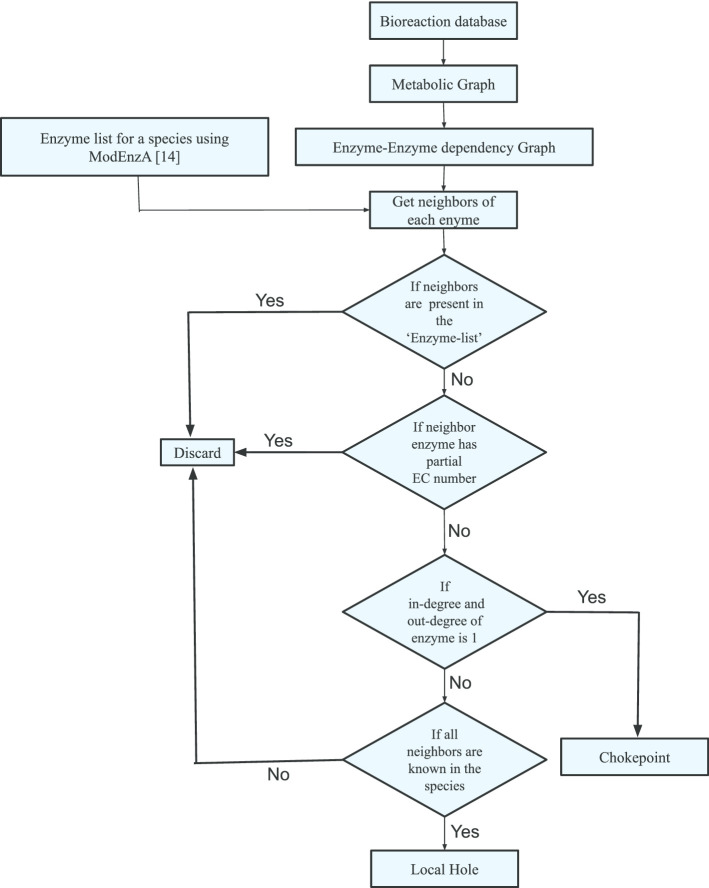
Schematic representation of the ‘Hole Finding Protocol’ to identify local holes and chokepoints in an organism. The figure shows a flowchart of the workflow for the identification of 'local holes' and ‘chokepoints’ in an organism using an enzyme–enzyme dependency graph of all known metabolic reactions. ModEnzA [14] is a profile HMM-based method used to scan the proteome of a given organism for the accurate classification of its enzymes

**Fig. 4:**
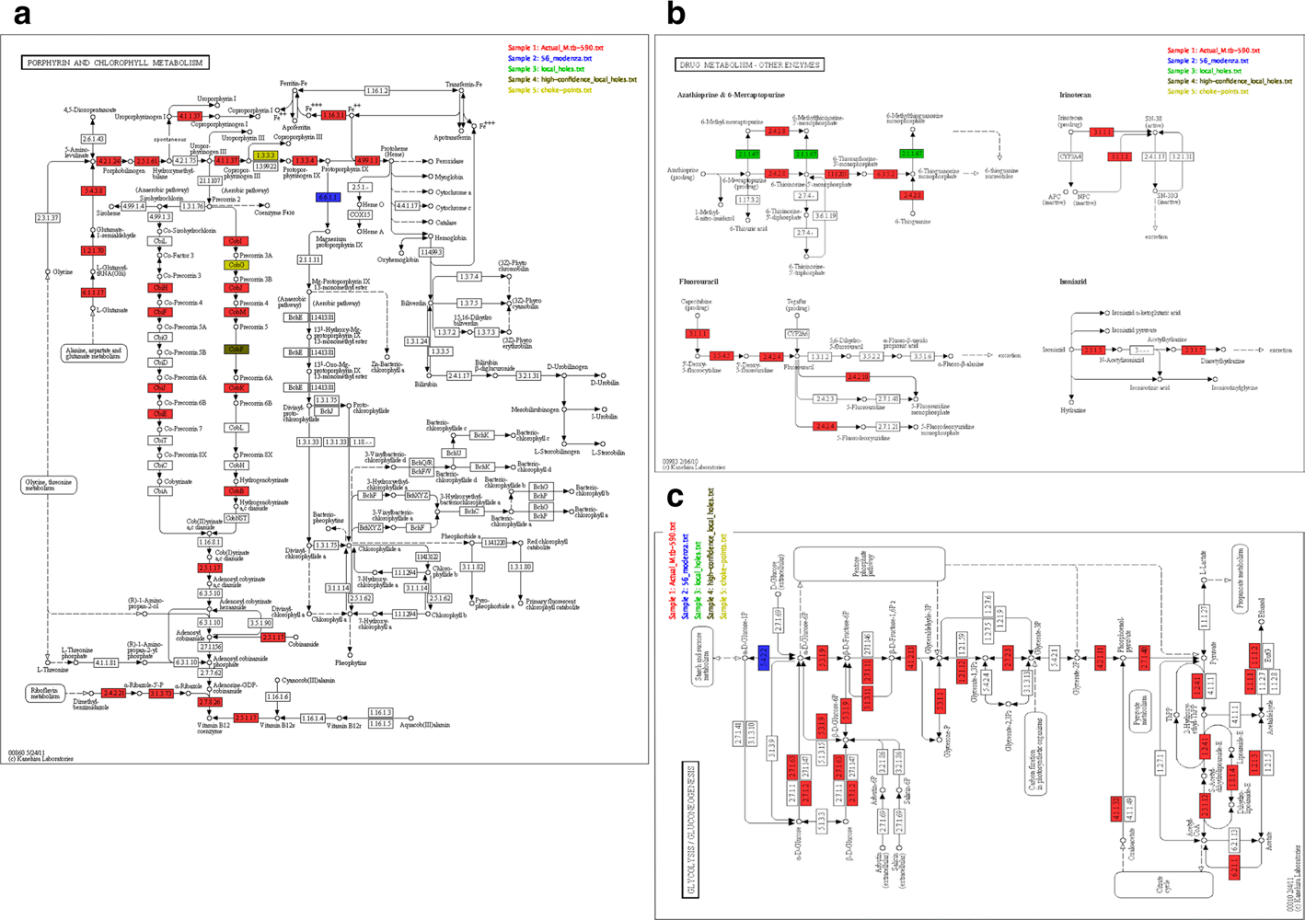
Mapping of known and predicted enzymes in *M. tb* on to KEGG pathways. **a**–**c** The mappings of some of these enzymes on the Porphyrin and Chlorophyll metabolism, Drug metabolism—other enzymes, and Glycolysis/Gluconeogenesis KEGG pathways respectively. The enzymes already annotated in * M. tb* are shown in Red, the enzymes predicted by the homology-based method ModEnzA are shown in Blue, the local holes in Green, the high-confidence local holes in Brown while the choke points are depicted in Yellow

The proposed protocols were tested in an incremental approach on *M. tb* as a case study. As shown in Fig. 2, we identified 56 novel predictions in *M. tb* (see Additional file 3: Supplementary file 1), using ModEnzA along with the 590 enzymes which are already reported in the ENZYME database (31 January 2018 release) for *M. tb*. We mapped the enzymes on to different pathways using the program FROMP [26] which maps and visualizes enzyme annotations onto the KEGG metabolic pathways and Gene Ontology terms. The known 590 *M. tb* enzymes (ENZYME database) map to 118 different KEGG pathways; the 56 novel enzymes predicted using the ModEnzA map to 43 pathways. These enzymes predicted by the homology-based method were then used to identify additional putative enzymes using the non-homology based method introduced in this study, ‘Hole finding protocol’, and shown in Fig. 3. Using the proposed method; we predicted 87 missing enzymes/holes in *M. tb* of which 76 are putative local holes, 8 choke points, and 3 high confidence local holes which map to 22, 5, and 5 different pathways respectively (Additional file 2: Supplementary Table 1). One of the examples of the mapping done for visualization using FROMP [26] is shown in Fig. 4.

**Table 1:** Predicted local holes and Chokepoints (shown in bold) in *M. tb* using the ‘Hole finding protocol’

Predicted hole/missing enzymes	Known neighbors in *M. tb* from EE graph	Protein ID
3.1.3.37 (Sedoheptulose-bisphosphatase/carbon metabolism)	4.1.2.13 (Fructose-bisphosphate aldolase)	Rv0363c
	2.2.1.1 (Transketolase)	Rv1449c
	2.2.1.2 (Transaldolase)	Rv1448c
	2.7.1.11 (6-phosphofructokinase)	Rv3010c
2.1.1.152 (Precorrin-6A synthase/porphyrin and chlorophyll metabolism)	1.3.1.54 (Precorrin-6A reductase)	Rv2070c
	2.5.1.6 (Methionine adenosyltransferase)	Rv1392
	2.1.1.133 (Precorrin-4 C (11)-methyltransferase)	Rv2071c
2.3.2.10 (UDP-N-acetylmuramoylpentapeptide-lysine N(6)-alanyltransferase/ Peptidoglycan biosynthesis)	6.1.1.14 (Glycine–tRNA ligase)	Rv0041
	6.1.1.7 Alanine–tRNA ligase	Rv2555c
	6.3.2.10 (MurF synthetase)	Rv2157c
	6.3.1.2 (Glutamine synthetase)	Rv2222c
1.14.13.83 (Precorrin-3B synthase/porphyrin and chlorophyll metabolism)	2.1.1.131 (Precorrin-3B C(17)-methyltransferase)	Rv2066
	2.1.1.130 (Precorrin-2C (20)-methyltransferase)	Rv2066
1.3.3.3 (Coprogen oxidase/porphyrin and chlorophyll metabolism)	1.3.3.4 (Protoporphyrinogen oxidase)	Rv2677c
	4.1.1.37 (Uroporphyrinogen decarboxylase)	Rv2678c
3.6.1.40 (Guanosine-5-triphosphate, 3diphosphate diphosphatase/purine metabolism)	3.1.7.2 (Guanosine-3,5-bis(diphosphate) 3-diphosphatase)	Rv2583c
	2.7.6.5 (GTP diphosphokinase)	Rv2583c
3.6.2.2 (Phosphoadenylylsulfatase/sulphur metabolism)	3.1.3.7 (3′(2′),5′-biphosphate nucleotidase)	Rv2131c
	2.7.1.25 (Adenylyl-sulfate kinase)	Rv1286
4.1.1.21(Phosphoribosylaminoimidazole carboxylase/purine metabolism)	6.3.4.18 (5-(carboxyamino)imidazole ribonucleotide synthase)	Rv3276c
	5.4.99.18 (5-(carboxyamino)imidazole ribonucleotide mutase	Rv3275c
5.4.99.61(Precorrin isomerase/porphyrin and chlorophyll metabolism)	6.3.5.9 (Hydrogenobyrinic acid a,c-diamide synthase)	Rv2847c
	2.1.1.132 (Precorrin-6 methyltransferase)	Rv2072c
6.2.1.28 (DHCA-CoA ligase)	1.2.1.3 (Aldehyde dehydrogenase (NAD(+))	Rv0458
	2.3.1.16 (Acyl CoA C-acyltransferase)	
6.3.3.1 (Phosphoribosylformylglycinamidine cyclo-ligase/purine metabolism)	6.3.4.18 (5-(carboxyamino)imidazole ribonucleotide synthase)	Rv3276c
	6.3.5.3 (Phosphoribosylformylglycinamidine synthase)	Rv0803

The table lists the local holes and chokepoints in the first column, along with the known neighbors in *M. tb* in the second column and the protein ID for the known neighbors in the third column

## Identifying local holes and chokepoints in the *M. tb* genome

To identify missing enzymes (local holes) in the *M. tb* genome, the known and novel predicted enzymes (646) were mapped to the enzyme–enzyme dependency graph (EE graph) generated using the bioreaction database [27]. The EE graph contains enzymes as nodes and edges representing a shared metabolite in a directed graph. For each of the enzymes in the list, the first degree of the metabolic neighborhood was identified. If the neighbor was already known in *M. tb* or it was an incomplete enzyme (enzymes having partial EC number) it was excluded from further analysis.

We found that the 646 *M. tb* enzymes had 1650 neighbors for which the in-degree (all the edges coming on the vertex) and out-degree (all the edges going out of the vertex) were identified. Each of these 1650 enzymes has at least one neighbor which is known in *M. tb*. As a high confidence filter, we only considered a node as a potential hole/missing enzyme when all the immediate neighbors for that node were present in *M. tb*. It was discovered that there are 87 such enzymes where all the neighbors were known in *M. tb*. Of these 87, 3 enzymes had all the neighbors (where there are at least one in-degree and one out-degree, and the sum of both is greater than 2) known in *M. tb*. These are predicted as ‘high confidence local holes’ in *M. tb* because they show connections with other known *M. tb* proteins and hence should be present in the pathogen. These are listed in Table 1 along with the listing of their known *M. tb* neighbors.

The remaining enzymes were further analyzed to identify probable chokepoints. The chokepoints were identified based on two criteria, first, both the neighbors are known in *M. tb*, and second that both the neighbors are different enzymes, i.e., it should not be a loop in the graph. Out of these 84 enzymes, 8 enzymes had both the neighbors known in *M. tb* while the remaining 76 enzymes had either in-degree or out-degree all of which are known enzymes in * M. tb*. These were also considered to be putative missing enzymes in *M. tb* and are listed in Additional file 4: Supplementary file 2. The 8 enzymes predicted as chokepoints are also tabulated in Table 1, along with the two nodes from the EE graph to which these enzymes were connected.

## Identifying candidate sequences for missing enzymes (holes)

**Fig. 5:**
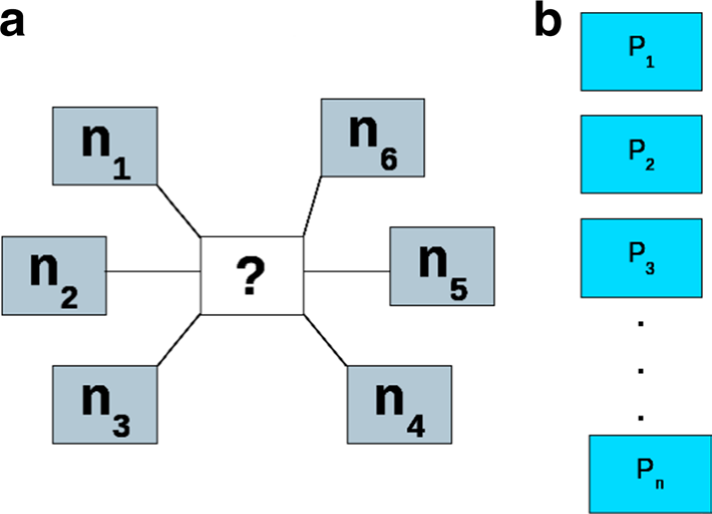
Schematic representation of a metabolic hole and candidate protein set. The figure shows **a** an unknown protein (?) surrounded by known neighbors n1–n6 and **b** a set of candidate proteins from the target organism which is its entire proteome except for the known neighboring proteins. For each neighbor, we find its interaction with all the candidate proteins. If protein P1 has an interaction score with n1, n2, and n4, then we combine these scores in a naive Bayes manner using Bayesian score integration as shown in the equation (see "Methods" ). All the candidate proteins with their respective scores are then sorted and the one with the highest score qualifies to perform the desired function

To identify a candidate protein for these missing enzymes, we have developed the ‘Hole filling protocol’ as shown in Fig. 5. We used a combined score of interactions (‘S’, see "Methods" section for the equation) for each protein obtained from the STRING database to rank the candidate sequences for a given hole (or missing enzyme). The performance of this method was assessed using the self-rank measure which estimates associations of the known metabolic neighborhood of an unknown enzyme with the candidate set of proteins as previously done [28]. A self-rank is defined as the rank of a known enzyme-encoding gene among the set of candidate proteins which contains all non-metabolic proteins as well as the known gene for which the rank is being estimated.

We first validated our hole-filling protocol using all the known metabolic proteins in *E. coli* which is a well-characterized model organism with an extensively annotated genome. All the known proteins from *E. coli* were ‘knocked out’ one protein at a time, and the candidate set along with the knocked out protein sequence was ranked based on the combined score, ‘S’. The known enzymes for *E. coli* were obtained by scanning the ModEnzA profiles across all the proteins of *E. coli*. There were a total of 459 metabolic proteins and 3375 non-metabolic proteins in *E. coli* (the actual number of metabolic and non-metabolic proteins is higher; in this study, we have only reported the number of proteins which had mappings to ENZYME CLASS (EC) numbers and STRING internal identifiers so that we can retrieve the combined score of association for the neighbors extracted from the EE graph, please refer to Additional file 1: Supplementary Fig. 1 for details about mapping the different IDs).

**Fig. 6:**
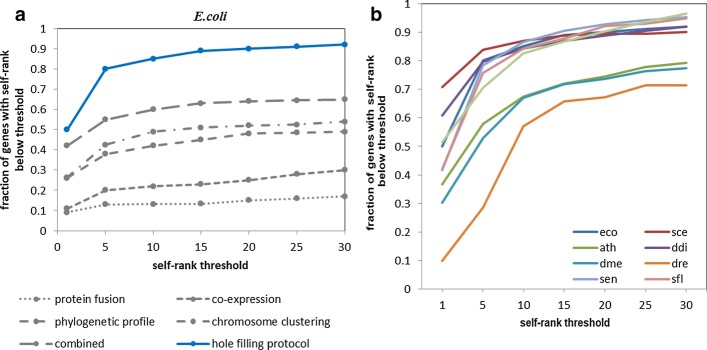
Comparison of the self-rank thresholds after in-silico enzyme knockouts. **a** The figure shows the performance of the ‘Hole Filling protocol’ on the *E. coli* genome (shown in the blue-colored curve) where the combined scores of functional associations from STRING were used to get the new functional association score. *Reference values for individual and combined association scores were digitized from Fig. 4 of Kharchenko et al. [28] for comparison. **b** Similar knockouts were performed for all the metabolic proteins from eight other genomes, *Saccharomyces cerevisiae (sce), Dictyostelium discoideum (ddi), Arabidopsis thaliana (ath), Drosophila melanogaster (dme), Danio rerio (dre), Salmonella enterica (sen), Shigella flexneri (sfl)* and *Vibrio cholerae (vch)*

Of 459 metabolic proteins, it was observed that for 85% of the cases the knockout protein is ranked within the top 10 of the candidate proteins (3375 non-metabolic proteins plus the knocked-out protein). We compared this result with a similar work which also reports the rank of proteins based on context-based methods of protein function prediction [28] using 351 metabolic proteins and 3352 candidate proteins set of *E. coli*. The comparison of our protocol with the various context-dependent methods used is summarized in Fig. 6a. The results from Kharchenko et al. [28], (Fig. 4 in their paper) show the rank of the *E. coli* proteins using individual methods of prediction like chromosomal clustering, phylogenetic profile, co-expression, protein fusion, and protein interactions and the rank after combining these methods. Their results ranged from 10% of the knocked-out genes falling within the top 10 candidate proteins using protein fusion to about 60% within the top 10 candidates using a combination of all methods. On the other hand, our protocol showed a 25% increase in prediction for the identification of known metabolic proteins within the top 10 candidates, using the combined score of the functional association from STRING and recalculated using Eq. 1 (Fig. 6a).

Additionally, we also used 8 other genomes to test the accuracy of our method. The selected genomes included *Saccharomyces cerevisiae, Dictyostelium discoideum, Arabidopsis thaliana, Drosophila melanogaster, Danio rerio, Salmonella enterica, Shigella flexneri*, and *Vibrio cholerae*. Figure 6b shows the performance of our method in knocking out a known metabolic protein and observing the self-ranks of the knocked out proteins. Except for the *Danio rerio* genome, all the other genomes showed that more than 50% or higher proteins were ranked in the top 5 ranks (Fig. 6b). After the in-silico validation on various genomes, we applied the ‘Hole filling algorithm’ on the predicted missing enzymes choke points and local holes, listed in Table 1, using all the non-metabolic and hypothetical proteins in * M. tb* as the candidate set. The set of matching proteins identified for the holes along with the rank is listed in Table 2. The rank of the protein is the highest rank of an uncharacterized protein. These matched proteins are the probable proteins that can be tested for the corresponding function experimentally. These chokepoints are important enzymes in a metabolic pathway representing potential drug targets in these pathways and may prove to be useful for the process of drug discovery.

**Table 2:** Predicted candidate protein from * M. tb* using the ‘Hole filling protocol’

Predicted missing enzyme	Predicted candidate protein	The rank of predicted candidate protein	Predicted GO molecular function
1.14.13.83 (Precorrin-3B synthase)	Rv2064	1	4Fe-4S cluster binding/metal ion binding/oxidoreductase activity
2.1.1.152 (Precorrin-6A synthase)	Rv2847c	2	Precorrin 2 dehydrogenase activity
1.3.3.3 (Coprogen oxidase/ Porphyrin and chlorophyll metabolism)	Rv2388c	1	Coproporphyrinogen III oxidase
6.2.1.28 (3-alpha,7-alpha-dihydroxy-5-beta-cholestanate–CoA ligase)	Rv0459	1	Uncharacterized protein
6.3.3.1 Phosphoribosylformylglycinamidine cyclo-ligase	Rv0809	1	Phosphoribosylaminoimidazole synthetase

The second column of the table shows the protein ID for the predicted candidate protein while the third column gives the rank of the prediction

## Discussion

Homology based automated enzyme prediction protocols provide ways for rapid annotation of completely sequenced genomes. Even though experimental verification of each of these predictions is time-consuming and often not feasible, protocols with increased accuracy and sensitivity, such as ModEnzA [14] can serve as a very important resource for generating reasonably good candidate proteins for experimental verification. The ModEnzA enzyme profiles built from an older version of the ENZYME database (09 July 2014) had predicted 213 novel annotations in *M. tb* of which 2.4.1.129, 3.1.1.3, 3.1.3.2, 3.4.16.4, 3.4.19.12 and 3.6.3.8 are now included as reviewed entries in *M. tb*. This highlights the strength of this method for the accurate identification of enzymes.

The manual analysis and literature survey suggests the importance of the new ModEnzA predictions in *M. tb*, made from the 31 January 2018 version (Additional file 3: Supplementary file 1, Tables 1 and 2), some of which are discussed here.

- 5.4.2.2 (Phosphoglucomtase) which is known to be one of the important enzymes in polysaccharide capsule formation is not among the list of annotated proteins in the ENZYME database for *M. tb*. but the protein scored by ModEnzA (I6Y2G3_MYCTU; E value = 4.00E−098) for this function is found to be experimentally verified by one of the research groups [29].
- 1.4.3.4 (Flavin containing monoamine oxygenase) is a part of amino acid metabolism (glycine, serine, and threonine metabolism) and catalyzes the conversion of aminoacetone into methylglyoxal. The same step can be catalyzed by another enzyme 1.4.3.21 (isozyme) and both of these are so far unknown in *M. tb*. We have found a significant hit for 1.4.3.4 in the *M. tb* proteome (AOFH_MYCTU; E value = 7.50E−98) which is a very good candidate protein for experimental validation.

These results serve to highlight the importance of ModEnzA in identifying or predicting enzymes that could serve as potential candidates for future validation.

In general, homology-based methods are the first choice of methods to be used for function assignment, but in the absence of any similarity with the well known/characterized sequences scope of these methods becomes limited. There are still many orthologs whose functions are not well defined, but they are conserved across multiple organisms [30, 31]. The chance of generating and propagating annotation errors is also very high when homology-based annotation methods are used. As yet, many different studies have quantified the inherent errors in the functional annotation of genomes and proteomes [32, 33]. These errors include some limitations of the underlying method itself [34], or errors in the annotations of the original database used for function assignment and errors when a new function is assigned to a homolog (gene/protein) as a result of evolutionary divergence [35].

Moreover, homology-based methods are biased towards exploring only the molecular functions of proteins rather than providing any information about the context of proteins within the cell. It is well established that proteins do not function in an isolated manner but interact with other biomolecules. Therefore, protein–protein interactions and protein networks are indeed very important to understand the function of a protein. As a result, a new class of in-silico methods came into the prominence to bring to light the cellular function of proteins. Unlike the homology-based methods, these methods rely on the context in which proteins are present within the cell and are known as non-homology/genome-context based methods [20, 36]. These methods comprise a plethora of functional associations between genes and proteins belonging to the same or different genomes [37] and provide an excellent alternative for proteins having no sequence homology to experimentally characterized homologs [38].

The non-homology based methods comprise different protocols based on protein–protein interactions. These interactions include both direct i.e. physical interactions or indirectly such as functional associations derived from methods like Gene Neighborhood, Gene Fusion, Co-occurrence, and Co-expression. Also, protein–protein interactions from various experiments, databases, and text mining approaches provide the necessary information. The metabolic neighborhood also provides a context for the co-occurrence of proteins which can be exploited for finding local holes (which have orthologs, but not in the genome understudy) and global holes or orphan enzymes (which have no known sequence associated with them). Methods based on sequence similarity cannot be applied to predict the function of such orphan enzymes. Genome context-based methods can be utilized for identifying sequences for these missing enzymes. The information about various context-based methods is available through various protein–protein interaction databases like BIND [39], BioGrid [40], KEGG [41], MIPS [42], ProLinks [43] and STRING [44].

Pioneering works in the incorporation of non-homology based method to identify functional links include a Bayesian technique to identify missing enzymes in metabolic pathways [45], identifying missing genes using the local structure of a metabolic network data [28], and extension of the Bayesian method to include genome context-based features for the identification of missing enzymes [46]. As compared to the hole finding and hole filling protocols discussed in this paper, which is a local approach to predict and identify orphan enzymes in a particular organism, a very similar global approach was recently developed to identify orphan enzymes across various genomes and metagenomes [18]. Both the methods use scores retrieved from STRING but they differ in the kind of scores used. We have used the combined functional association score (coming from various kind of shreds of evidence like the neighborhood, gene fusion, co-occurrence, coexpression, database, text mining, and homology-based scores) while the other method uses only neighborhood and co-occurrence from STRING in addition to a signature binding domain score and a pathway network score.

In this work, we highlight the importance of non-homology based methods utilizing the metabolic network context, to predict the function of protein when there is no significant sequence similarity. Such methods are of utmost importance to identify orphan enzymes in a metabolic pathway. We have developed methods (a) to identify missing enzymes in a pathway (Hole finding protocol) and (b) to associate protein sequences to the holes from a set of non-metabolic and hypothetical candidate sequences (Hole filling protocol) by using combined functional association scores retrieved from the STRING database. The identification of missing enzymes in a metabolic pathway requires information about the various reactions in that pathway which in turn is useful for the accurate reconstruction of metabolic pathways. There are various resources providing information for these reactions like KEGG, BKM-React [47], and the Ma-Zeng bioreaction database [27]. The data from the former two resources require a subscription to access while the latter is publicly available. This database was recently upgraded to a newer version and includes double the number of reactions.

A detailed biochemical experimental validation for each of the predictions is beyond the scope of this work. In context-based methods, the metabolic context of an enzyme is confined to its immediate neighbors both from a reaction base graph as well as an association graph derived from the STRING database. The STRING database, in particular, has been used frequently as a tool for inferring missing protein activities in genomes thereby extending or increasing the protein repertoire of organisms including pathogenic organisms [48, 49]. There have been recent instances in the literature of fitting the power-law distribution to PPI networks generated using STRING data [50, 51]. For a PPI network generated using a threshold of 0.9, only a fraction of the nodes (from the right tail of the distribution) fit the Power-law function [50]. In the other case, networks generated for 3 sets of differentially expressed genes (upregulated, downregulated, and total) using data from the STRING database version 10 with a confidence threshold of 0.4, all followed the Power-law with R2 values of 0.749, 0.859, and 0.836 respectively [51]. Although we have not analyzed the Power-law fit of the STRING network at a threshold of 0.7, the above evidence suggests that the network data is following Power-law distributions.

We have used the combined scores of functional associations from the STRING database and modified them to be used in an automated protocol for predicting missing enzymes. We carried out a mapping of the predicted enzymes, in this study, to KEGG pathway maps. The 3 predicted local holes map to three different pathways; EC 2.3.2.10 is a part of peptidoglycan biosynthesis, EC 2.1.1.152 belongs to Porphyrin and chlorophyll metabolism while EC 3.2.1.37 belongs to Carbon metabolism. Among these, cases like 2.1.1.152 (only one characterized sequence in enzyme database; P21636; COBF_PSEDE) where there are not enough well-characterized sequences, hole finding algorithms can provide contextual information which in turn could be useful for providing functional annotations. Moreover, since 2.1.1.152 is also known to be present in other strains of *Mycobacterium* [52] therefore it is highly likely to be present in the *M. tb* H37Rv genome used as a case study in this work and this supports our prediction which classifies this enzyme as a missing enzyme/local hole.

It has been discussed in the literature that the Cobalamin biosynthesis pathway is likely to be present in *M. tuberculosis* [53]. The four enzymes which are required for the conversion of precorrin-3A to precorrin-6A are 1.14.13.83 (Precorrin-3B synthase, CobG) yielding precorrin-3B. This step is followed by three successive methylation steps which introduce a methyl group at C-17, C-111, and C-1. These three methylation steps are catalyzed by enzymes 2.1.1.131 (Precorrin-3B C(17) methyltransferase, CobJ), 2.1.1.133 (Precorrin-4 C(11) methyltransferase, CobM) and 2.1.1.152 (Precorrin-6A synthase, CobF) to yield precorrin-4, precorrin-5, and precorrin-6A respectively. Of these, two enzymes 2.1.1.131 and 2.1.1.133 are known to be present in *M. tuberculosis* and therefore it is highly likely that the other two enzymes should also be present in the * M. tb*. We noticed that the protocol described in this study identifies the rest two enzymes 1.14.13.83 and 2.1.1.152 as missing enzymes (holes) and interestingly predicts 1.14.13.83 as a choke point and 2.1.1.152 as a local hole, and this makes sense as the first step in the conversion of precorrin-3A to precorrin-6A is catalyzed by 1.14.13.83 (usually the source and sink reactions in a pathway are unique). The ‘hole filling protocol’ ranked the proteins Rv2064 and RV0511 as the top-ranked candidates for the enzymes 1.14.13.83 and 2.1.1.152, respectively. A more detailed analysis of these proteins tells us that both are unreviewed entries in UniProt with only electronic annotation (which is an annotation by similarity and does not include any experimental evidence to support the annotation). Rv2064 is annotated as Precorrin-3B synthase and which matches with the functionality of the enzyme 1.14.13.83, however, since no experimental evidence is available, this protein is not included in the ENZYME database. We compared the only sequence belonging to the EC 1.14.13.83 in the ENZYME database P21637 (COBG_SINSX) to Rv2064 (candidate protein with rank 1) and found that they both share the presence of the same Pfam domain (PF03460: NIR_SIR_ferr﻿) providing additional evidence for the functionality for the candidate protein Rv2064. Similarly, for Rv0511 which is ranked 1 for the function, EC 2.1.1.152, Precorrin-6A synthase, the presence of the same Pfam domain TP_methylase (PF00590) supports the prediction using our method. These annotations, if experimentally validated, could complete the presence of the chain of enzymes required for the conversion of precorrin-3A to precorrin-6A. The prediction also reflects the ability of our method to pick the best possible candidate for a missing enzyme.

The eight predicted chokepoints are mapped to three pathways; 6.3.3.1 (Phosphoribosyl formylglycinamidine cyclo-ligase), 4.1.1.21 (Phosphoribosylaminoimidazole carboxylase) and 3.6.1.40 (Guanosine-5′-triphosphate, 3′-diphosphate diphosphatase) map to ‘Purine metabolism’; 1.14.13.83 (Precorrin-3B synthase), 5.4.99.61 (Porphyrin isomerase) and 1.3.3.3 (Coproporphyrinogen oxidase) belong to ‘Porphyrin and chlorophyll metabolism’; 3.6.2.2 (Phosphoadenylylsulfatase) is a part of sulfur metabolism, reduction, and fixation while 6.2.1.28 (DHCA-CoA ligase) do not map to any KEGG pathway map using the FROMP program [26]. Available ModEnzA profiles for these chokepoints (except for 1.14.13.83, having only one sequence and 3.6.2.2, 6.2.1.28 which are global holes having no known sequence in any organism, thereby having no ModEnzA profiles) do not give any significant hit.

The enzyme 4.1.1.21 (Phosphoribosylaminoimidazole carboxylase) is unknown in *M. tb* but is directly connected to 6.3.4.18 (5-(carboxyamino) imidazole ribonucleotide synthase) and 5.4.99.18 (5-(carboxyamino) imidazole ribonucleotide mutase)) both of which are known in *M. tb*. The protocol predicted 4.1.1.21 as a missing enzyme but on a closer look, we found that the reaction catalyzed by this enzyme occurs in vertebrates during purine biosynthesis but in the case of bacteria two other enzymes are required to carry out the same reaction namely EC 6.3.4.18 and EC 5.4.99.18. This suggests that 4.1.1.21 should not be considered as a missing enzyme in *M. tb* due to the presence of the other two enzymes 6.3.4.18 and 5.4.99.18, and such cases are exceptions to our protocol.

Overall, these findings indicate the importance of context-based methods to identify enzymatic activities in absence of significant sequence similarity. The predicted candidates from the hole filling approach listed in Table 2 could be used as candidates for increasing the protein repertoire of pathogenic microorganisms upon future experimental validation. Since chokepoint enzymes by definition have no other alternative pathways that could circumvent their function, they are also prime candidates for evaluation as drug targets. In general, our method is useful for prioritizing the candidate proteins for a missing metabolic protein from any genome to be validated experimentally. Additional computational analysis of candidate sequences including domain analysis, secondary structure prediction, etc. might be necessary to assess the top-ranked candidate sequences. However, the method could still pick up false positives as we do see in Fig. 6b that not all the known metabolic proteins give a self-rank of 1 which would have been the ideal case. As the STRING database and the database of metabolic reactions continue to grow and accumulate information, the accuracy of protocols such as the ones described in this manuscript is expected to improve further.

## Conclusions

In this work we proposed, Hole finding protocol, for the identification of local holes in an organism and a downstream protocol, Hole filling protocol, for the identification of candidate proteins for the predicted metabolic holes. The Hole filling protocol uses a scoring scheme that integrates the combined functional scores from the STRING database. Our results signify the importance of such automated protocols for accurately annotating and extending the protein repertoire of pathogenic organisms to identify novel candidates for drug targets which could be experimentally verified in the future. We conclude that the use of non-homology based methods complement the homology-based approaches when used together in conjunction and result in a more complete annotation.

## Methods

### Enzyme identification in the *M. tb* genome

For any given species, a list of known enzymes can be retrieved using the in-house developed method ModEnzA [14]. The enzyme profiles of the latest ENZYME database (26 February 2020) were built which represents a comprehensive resource for the accurate identification of enzymes in any organism. These profiles were used to scan the *M. tb* proteome.

## Hole finding protocol

The updated Ma-Zeng bioreaction database [27], which uses KEGG Ligand [39] and Brenda [54] for compiling the information, contains 6851 reactions catalyzed by a set of 3525 enzymes. In this study, we used the bioreaction database to create a metabolic graph where reactants and products are the nodes, and the enzyme catalyzing that particular reaction represents an edge. This graph was used to create an enzyme–enzyme dependency graph (EE graph) where two enzymes E1 and E2 are connected if the product of the reaction catalyzed by E1 is the substrate for the reaction catalyzed by E2. The resulting graph represents a superset of connections for enzymes from all the known reactions and is used to identify local holes and choke points in a given organism. The protocol developed is shown in Fig. 3. To identify potential missing enzymes (local holes) the annotated enzymes were mapped onto the EE graph to identify their neighboring counterparts (‘neighbors’ from the CPAN package ‘Graph’ was used to identify the neighbors for each of the enzymes). The resulting neighbors were mapped back to the original enzyme list to discard all those enzymes which were already known in the given organism. Besides, any neighboring enzymes having partial EC numbers or having incomplete information were discarded from further analysis. The in-degree and out-degree were calculated for all the neighboring enzymes; if there is only one in-degree and one out-degree for a given node and both of these are known enzymes then the node was predicted as a chokepoint based on the unique connectivity of the node. Whereas, if there is more than one connection to the input node and all of these are known in the given organism then the node was predicted to be a local hole/missing enzyme in the organism.

## Hole filling protocol

The identification of candidate protein sequences for the predicted missing enzymatic activities (holes) is based on the hypothesis that proteins present in similar pathways are often functionally interacting. Therefore, the known proteins which form the neighborhood of an unknown protein in the same pathway should have very high protein–protein association scores with the candidate sequences. These scores can then be used to rank the candidate proteins to select the best probable proteins for the function. The combined functional association scores were retrieved from STRING (v11.0) which gives higher confidence of prediction than the individual scores. This score was used for all the known interactions between neighbors of an unknown protein (missing enzyme) and a set of candidate proteins.

Given a pathway hole (unknown protein) in a metabolic pathway, we can identify its known neighborhood from the EE graph. As shown in Fig. 5, assume that the unknown protein in the center has six neighbors, the function of which is known. There are ‘n’ proteins in the target proteome which are either not part of the metabolism i.e. the non-metabolic proteins or are proteins with unknown function. These represent the set of candidate proteins. The idea is to use the protein–protein association scores of the neighborhood proteins with the candidate proteins and then rank the candidate sequences based on the scores. STRING offers different levels of confidence at different cutoffs, the highest cutoff being 0.9, followed by 0.7 which are the high confidence hits, followed by 0.5 which are the medium confidence hits, and finally, 0.150 which is the low confidence threshold. To reduce the noise, we have only used the ‘high confidence cutoff 0.7′ for finding the interactions between the known neighbors and the candidate protein set. All scores which are greater than a probability of 0.7 are included in our analysis.

A single candidate protein may interact with multiple neighbors, each pairwise interaction contributing an individual score. In this case, we proposed to combine these pairwise scores in a simple expression of individual combined scores of associations:

$${\text{S}} = {1} - \prod {\text{i}}\left( {{1} - {\text{s}}_{{\text{i}}} } \right)$$

where s_i_ is an individual combined score of a candidate protein with a neighboring protein of the missing enzyme iterated over ‘i’ pairs. ‘S’ is the final combined score. It may be noted that the same expression is employed by the STRING database to combine individual channel scores to generate the combined score for a pairwise interaction [55].

In the ideal case, we expect to get a rank of ‘1' for a hypothetical protein which could be used as the best possible protein for the desired function. A rank of ‘1' means that the corresponding protein shows maximum association with the known neighborhood of the missing enzymes. Therefore, this protein can be tested for the function of the missing enzyme.

## Supplementary information

**Additional file 1:** Supplementary Figure 1 Schematic representation of the mapping protocol: EC number to STRING internal identifier.

**Additional file 2:** Supplementary File 1 List of 56 novel enzyme prediction in Mycobacterium tuberculosis H37Rv genome using the method ModEnzA [14].

**Additional file 3:** Supplementary File 2 List of 76 putative missing enzymes that have either in-degree or out-degree, all of which are known in Mycobacterium tuberculosis H37Rv genome.

**Additional file 4:** Supplementary Table 1 A table showing the mapping of predicted 87 missing enzymes/holes (76 putative local holes, 8 choke points, and 3 high confidence holes) to different pathways using the method FROMP [26].

## Data Availability

The dataset and scripts for ModEnzA and the new methods developed are available via GitHub from the following links: https://github.com/dhwanidesai/ModEnzA. https://github.com/dhwanidesai/EnzymeHoleFindFill.

## Abbreviations

*M. tb*: *Mycobacterium tuberculosis*
HMM: Hidden Markov model
PSSM: Position-specific scoring matrix
