# Supplementary figures and images for: Implementation of homology based and non-homology based computational methods for the identification and annotation of orphan enzymes: using Mycobacterium tuberculosis H37Rv as a case study
Source: BMC Bioinformatics. 2020 Oct 19;21:466. doi: 10.1186/s12859-020-03794-x (PMC7574302; doi:10.1186/s12859-020-03794-x)

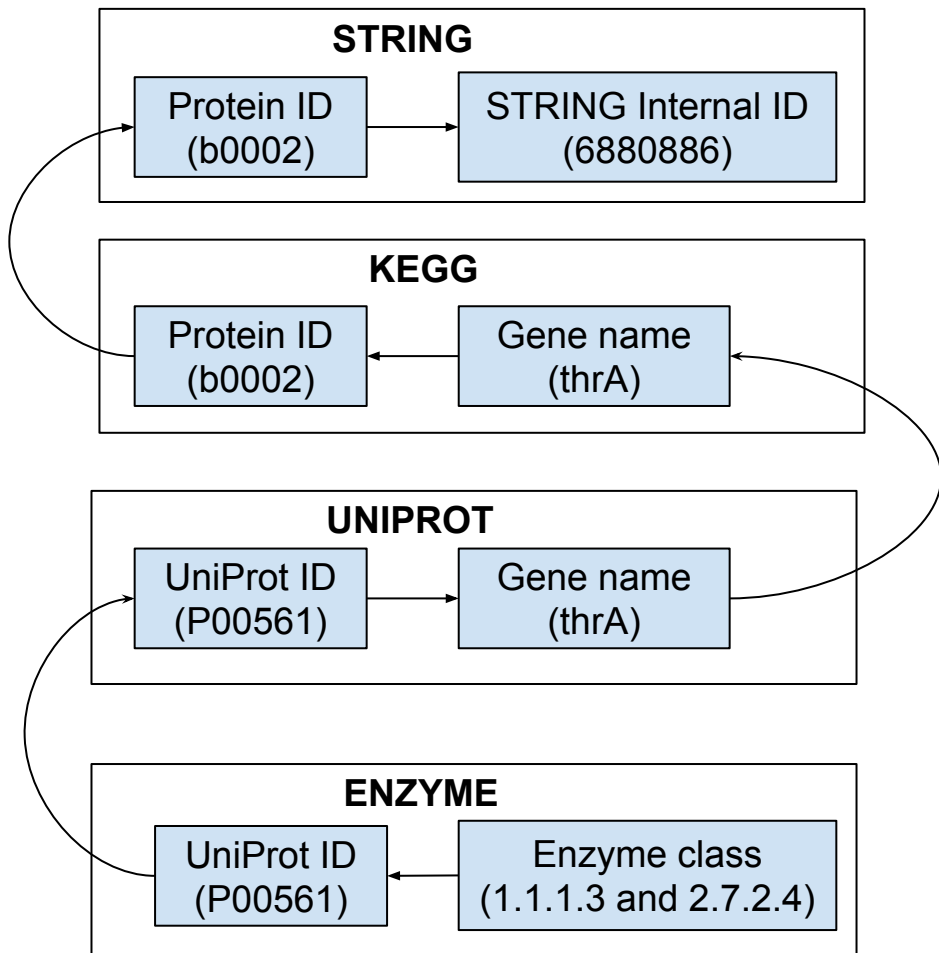

Supplement: Supplementary file 1 — Additional file 1: Supplementary Figure 1 Schematic representation of the mapping protocol: EC number to STRING internal identifier. [file 12859_2020_3794_MOESM1_ESM.pdf]
